# Conditional Variational Autoencoder for Functional Connectivity Analysis of Autism Spectrum Disorder Functional Magnetic Resonance Imaging Data: A Comparative Study

**DOI:** 10.3390/bioengineering10101209

**Published:** 2023-10-16

**Authors:** Mariia Sidulova, Chung Hyuk Park

**Affiliations:** 1 Department of Biomedical Engineering, School of Engineering and Applied Science, The George Washington University, Washington, DC 20052, USA; sidul001@gwmail.gwu.edu; 2 Department of Computer Science, School of Engineering and Applied Science, The George Washington University, Washington, DC 20052, USA

**Keywords:** fMRI, functional connectivity, autism spectrum disorder, autoencoders, conditional variational autoencoders

## Abstract

Generative models, such as Variational Autoencoders (VAEs), are increasingly employed for atypical pattern detection in brain imaging. During training, these models learn to capture the underlying patterns within “normal” brain images and generate new samples from those patterns. Neurodivergent states can be observed by measuring the dissimilarity between the generated/reconstructed images and the input images. This paper leverages VAEs to conduct Functional Connectivity (FC) analysis from functional Magnetic Resonance Imaging (fMRI) scans of individuals with Autism Spectrum Disorder (ASD), aiming to uncover atypical interconnectivity between brain regions. In the first part of our study, we compare multiple VAE architectures—Conditional VAE, Recurrent VAE, and a hybrid of CNN parallel with RNN VAE—aiming to establish the effectiveness of VAEs in application FC analysis. Given the nature of the disorder, ASD exhibits a higher prevalence among males than females. Therefore, in the second part of this paper, we investigate if introducing phenotypic data could improve the performance of VAEs and, consequently, FC analysis. We compare our results with the findings from previous studies in the literature. The results showed that CNN-based VAE architecture is more effective for this application than the other models.

## 1. Introduction

Autism Spectrum Disorder (ASD) is a neurodevelopmental disorder/condition in which individuals experience difficulties in social communication and interaction and exhibit limited or repetitive behaviors and interests. Additionally, autistic individuals may have alternative learning styles, movements, and attention patterns [1]. Several studies have consistently shown that ASD is more commonly found in males than females, with an approximate ratio of 3 to 1 [2]. One of the approaches used to investigate neurodivergence associated with ASD is Functional Connectivity (FC) analysis of functional Magnetic Resonance Imaging (fMRI) data. FC analysis helps to examine statistical dependence between the activity of different brain regions based on their blood oxygenation levels measured by fMRI [3]. Hence, FC represents the extent to which various brain regions exhibit synchronized activity over a period of time, which is commonly believed to be representative of the structural and functional organization of the brain [3].

Functional Connectivity (FC) studies in ASD have led to the development of two main theories about the connectivity of the brains of individuals with ASD: under-connectivity and over-connectivity [4]. Under-connectivity is defined as a decrease in brain activity between brain regions compared to a neurotypical population [5]. Conversely, over-connectivity is understood as higher statistical correlations between different areas of the brain appearing in affected individuals compared to unaffected individuals [6]. Finally, as more recent studies indicate, it is more likely that both over- and under-connectivity patterns are present in the brains of individuals with ASD [4]. Traditional methods for FC analysis include Seed-Based Correlation Analysis (SCA) [7], Independent Component Analysis (ICA) [8], graph-theory-based analysis [9], clustering-based approaches [10], dynamic connectivity analysis [11], Granger causality analysis [12], and dynamic causal modeling [13]. While these approaches have helped uncover neurodivergent patterns in fMRI data, they entail certain limitations, such as inherent biases or limited interpretability. Several inconsistencies have been reported in studies using these methods when examining functional connectivity patterns in fMRI in ASD. The discrepancies are mainly attributed to the varied age and sex compositions within the study samples and the diverse nature of ASD [4]. Notably, an apparent trend of under-representation of females with ASD in FC studies of fMRI can be seen [4]. Limited interpretability arises from technical constraints inherent to each of these methods, which are comprehensively examined in Section 2.1.

To address the issues of limited interpretability and under-representation, we propose a novel approach to FC analysis of fMRI data using Variational Autoencoders and Conditional Variational Autoencoders. The Variational Autoencoder (VAE) is a deep generative model that learns to encode data into a low-dimensional latent space and then decodes low-dimensional features back to the original data [14]. The Conditional Variational Autoencoder (CVAE) is an extension of the standard VAE, which incorporates conditional information, such as additional class features or attributes, into the generative model to enable targeted data synthesis [15]. This study examines the application of three different VAE architectures for FC analysis for individuals with ASD. We then apply phenotypic data to VAEs in an attempt to reduce sex-related bias. For a more quantitative and structured analysis, we have employed three commonly used VAE architectures in the fMRI domain: Convolutional Neural Network (CNN), Recurrent Neural Network (RNN), and a hybrid model combining CNN and RNN in parallel. Our evaluation of the VAE and CVAE includes comparing the performance in the reconstruction of neurotypical samples and the efficacy in conducting FC analysis for fMRI samples of individuals with ASD. Our evaluation compares the identified FC divergences between female and male populations for both VAE and CVAE. We aim to provide a structural and systemic investigation with diverse AE architecture variations in the fMRI domain, specifically addressing the issues of dynamic processing of highly complex brain imaging data and sex under-representation with statistical modeling.

This paper is structured as follows: we first discuss the pertinent literature on traditional FC methods and the utilization of VAEs and CVAEs in the fMRI domain. Additionally, we provide a concise overview of previously investigated FC divergences in ASD. Subsequently, in Section 3, we introduce the dataset, explain the data preprocessing techniques employed, elaborate on the VAE and CVAE architectures utilized, and detail our FC analysis approach. In Section 4, we present our findings and the results of our experiments, and in Section 5, we draw comparisons between our findings and those of previous studies. In Section 6, we summarize our findings and discuss possible future directions.

## 2. Related Works

### 2.1. Traditional Approaches to FC Analysis

Various methods have been developed to examine brain functional connectivity using fMRI data [16], which includes Seed-Based Correlation Analysis (SCA) [7], independent component analysis (ICA) [8], and graph-theory based analysis [9]. SCA involves selecting a Region of Interest (ROI) and computing its correlations with other brain regions over time series. High correlations indicate over-connectivity, and low correlation under-connectivity. However, SCA can potentially introduce bias due to ROI selection, overlooking important connectivity patterns outside the chosen regions [17]. On the other hand, ICA is a data-driven, multivariate method that decomposes fMRI data into spatially independent components, each representing a unique spatial pattern associated with a distinct time course [8,18]. ICA has been applicable in revealing lower-level spatial and temporal patterns in brain connectivity. Nevertheless, the drawback of ICA analysis is that the signal from a single brain region may appear in multiple components within lower-dimensional space, complicating the identification of high-level correlations [4]. Graph theory provides a framework for investigating local and global connectivity patterns. However, effectively capturing the temporal dynamics inherent in fMRI data presents a significant challenge. More advanced traditional approaches to Functional Connectivity Analysis (FC) include clustering-based approaches [10], dynamic connectivity analysis [11], Granger causality analysis [12], and dynamic causal modeling [13]. Most studies using traditional methods have focused on male fMRI data with ASD, and there has been a lack of research specifically exploring females with ASD. When the dataset is imbalanced, SCA, ICA, and graph-based analyses face several challenges. For example, SCA is often used to compare connectivity patterns between different subgroups; thus, an imbalance in the studied data can influence the statistical power and robustness of the comparisons. In ICA, while the analysis is not inherently affected by class imbalance, subsequent classifiers that use ICA-derived features may favor the majority class, affecting classification performance. In graph-based methods, graph construction could also be hindered by the greater presence of certain populations. Therefore, there is a need for an approach that encompasses both the spatial and temporal distribution of the data and is robust to under-representations in the dataset.

### 2.2. Application of VAEs in fMRI Domain

To address some of the challenges mentioned in Section 2.1, recently, there has been a surge in the utilization of VAEs to identify brain connectivity patterns within affected populations or fMRI signal patterns related to specific tasks. VAEs offer the advantage of allowing for the studying of both low- and high-level features of fMRI data, setting them apart from techniques such as ICA and SCA. Several papers used VAEs to extract meaningful features to classify the data [19,20,21]; some studies also researched the abilities of VAEs to identify task-related activities [22,23], and finally, some utilized VAEs for FC analysis of the fMRI data [24,25].

The most closely related to our works is the paper by Zuo et al., in which the researchers utilized a disentangled VAE to identify structural and functional connectivity differences between control, individuals with early mild cognitive impairment (MCI), and individuals with late mild cognitive impairment [24]. Using a graph convolutional VAE, researchers have identified under- and over-connectivity patterns associated with the progression of MCI. Likewise, another study by Choi et al. applied a Deep Neural Network (DNN)-based VAE to analyze connectivity patterns in ASD [25]. The study has also presented under- and over-connectivity patterns correlated with the full-scale IQ scores.

A considerable number of encoder and decoder architectures have been studied in the application of fMRIs, which vary depending on the main objective of the application. However, the most common architectures include convolutional layers (CNN), recurrent layers (RNN), and a combination of the two in sequence and parallel. CNN layers have proven to be helpful in identifying spatial correlations; however, the temporal patterns of the decoded data are not meaningful since the convolution is not capable of capturing the temporal dynamics. And vice versa, recurrent layers have shown to have better temporal feature extraction, but spatial patterns could not be well preserved. Therefore, we believe that there is a need to evaluate different model architectures.

### 2.3. Application of CVAEs in fMRI Domain

**Figure 1:**
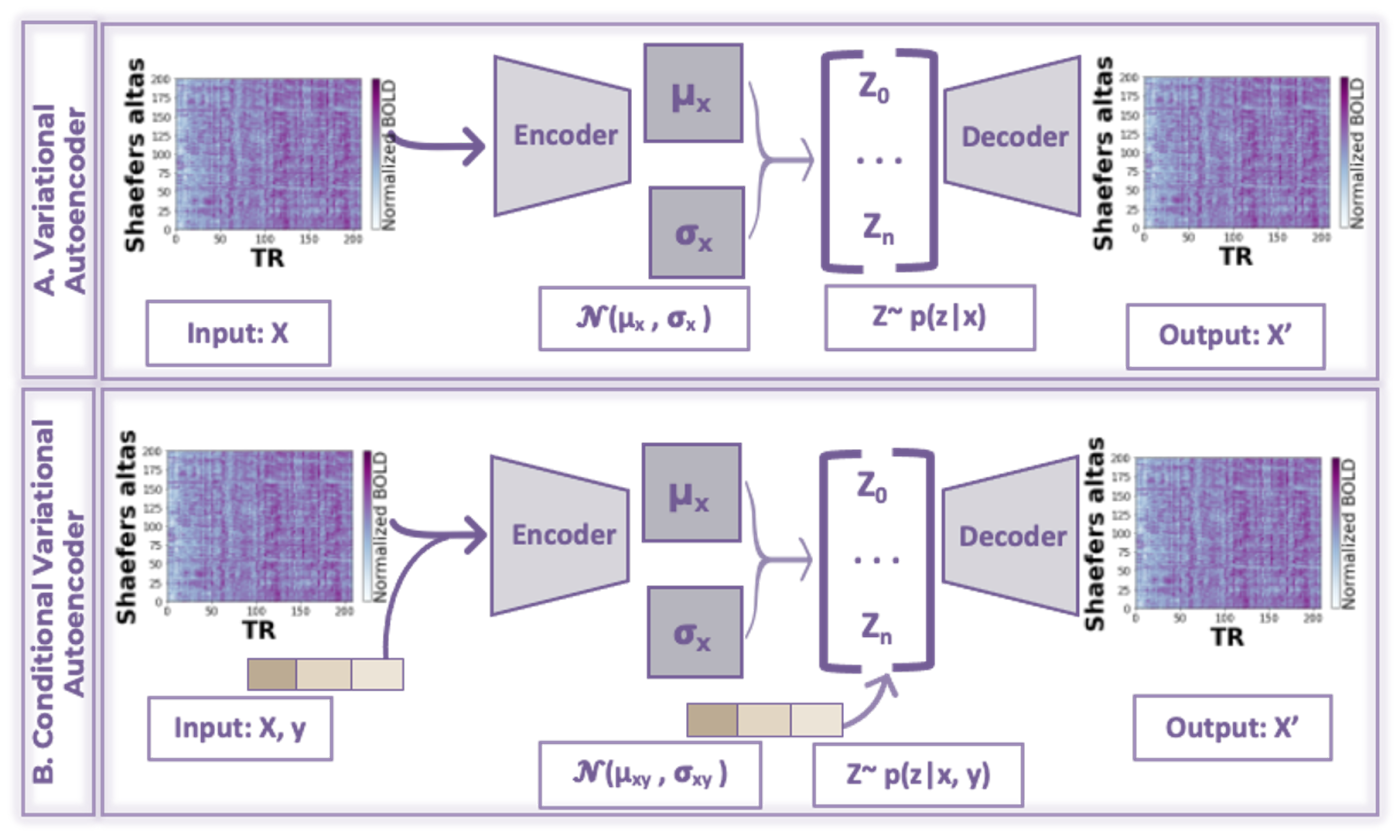
Summary of the difference between VAE and CVAE. In CVAE, both the encoder and decoder part receive conditional attributes; in our study, it is an embedding consisting of age, sex, and group label.

Conditional Variational Autoencoders (CVAEs) are an extension of the VAEs that incorporate additional information into the generative model [15]. The generative process in a CVAE is improved by considering additional information, such as class labels, attributes, or any other relevant data. Conditional variables are then passed into both the encoder and decoder parts of the VAE (Figure 1). Therefore, the encoder takes the input data and associated conditional variables and maps them to a distribution in the latent space. The decoder then uses the sampled latent distribution from the encoder along with the conditional variables to reconstruct the input data point. By adding additional information to the generation process, CVAEs allow for more targeted and controlled data generation. In the context of fMRI imaging, CVAEs have been used for image synthesis and data augmentation [26], brain image segmentation [27], classification [28], and connectivity network detection [29]. The most closely related to our study is the study by Wang et al., which used adverse CVAE to identify high-level neurodivergent patterns associated with Alzheimer’s Disease (AD) in fMRI data [30]. Researchers have demonstrated that applying conditions to the network helps reduce the effect of age and sex bias in the latent vectors. Another paper that used CVAE is the study by Gao et al., where researchers integrate age and sex attributes through an attention mechanism that optimizes VAE for the classification of brain connectivity from fMRI data of individuals with attention-deficit/hyperactivity disorder from multiple sites [31]. The study showed that phenotypic information has improved learning discriminative embedding and helped identify affected brain regions functionally by reconstructing the latent features.

### 2.4. Functional Connectivity in ASD

The most commonly studied brain networks in ASD include Default Mode Network (DMN), limbic, visual, somatomotor, and salience networks. The regional components of each of these networks have a tendency to slightly change study by study. The DMN is a large-scale brain network that is most active during rest periods or when the mind is wandering [32]. It is involved in various cognitive processes such as self-thinking, episodic memory recovery, and social cognition [32]. In most studies, the DMN includes regions such as the medial prefrontal cortex, the posterior cingulate cortex, and the medial temporal lobes [4]. The limbic network is a group of interconnected structures that play a critical role in emotion, motivation, and memory processing [33]. The limbic network is closely associated with the management of emotional responses, the processing of reward and punishment, and the formation and recovery of memories. Key structures in the limbic system include the amygdala, hippocampus, and cingulate gyrus [34]. The visual network is responsible for processing visual stimuli, and its nodes are located primarily in the occipital lobe [35]. The somatomotor network is involved in the planning, enactment, and management of voluntary movements [3]. It includes the primary motor cortex, the supplementary motor area, and the primary somatosensory cortex, all located in the frontal and parietal lobes. Finally, the salience network is a large-scale brain network that is involved in catching and focusing attention to relevant internal and external stimuli [36]. Key regions within the salience network include the anterior insula and the dorsal anterior cingulate cortex [37].

Previous findings suggest that under-connectivity between various brain networks is associated with social impairments and deficits observed in ASD. Most under-connectivity patterns were associated with DMN, including decreased interconnectivity between DMN-limbic, DMN-visual, and DMN-somatomotor. For example, in the study by Abrams et al., the researchers reported under-connectivity between DMN (pSTS with orbitofrontal, temporal lobe) and limbic networks (amygdala), suggesting that ASD individuals experience a less pleasant response to human voice processing [38]. Under-connectivity between the DMN (Precuneus (PrC)) and the visual cortex has also been previously reported [39]. However, the study reported that this under-connectivity pattern was not found to be related to socio-behavior deficits. Finally, under-connectivity between DMN and several regions in somatomotor has also been reported in multiple studies [40,41].

Over-connectivity patterns are primarily associated with salience networks. For example, a study by Green et al. has demonstrated the over-connectivity of the salience network with sensory processing areas, such as the visual and limbic networks, in individuals with ASD. It is believed that this over-connectivity may contribute to heightened responsiveness to irrelevant stimuli and deficits in social interactions [42]. DMN-salience network was shown to have higher interconnectivity in ASD subjects compared to TD in work by Yerys et al. [40], which has been hypothesized to be attributed to the ability to switch between intra-person and extra-person processing.

A handful of studies specifically looked into the difference between female and male functional connectivity. One of the few studies of specifically sex-related differences revealed that commonly associated DMN hypoconnectivities are primarily present in male populations [43]. Increased connectivity in the female population compared to males has also been supported by the studies by Lawerence et al. [44] and Smith et al. [45].

## 3. Materials and Methods

### 3.1. Dataset

**Figure 2:**
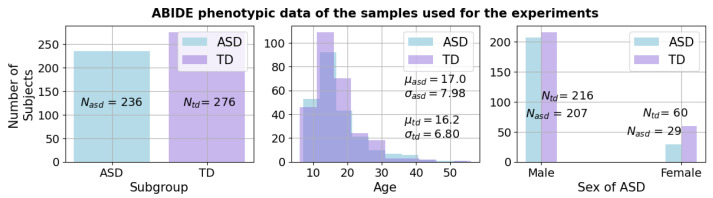
Summary of phenotypic data is presented. In particular, the number of male samples is higher than that of females in both subjects with typically developing ASD and subjects with ASD.

The ABIDE-I (Autism Brain Imaging Data Exchange) dataset is a publicly available, large-scale collection of resting-state fMRI data of individuals with ASD [46]. The ABIDE-I dataset consists of 1035 rs-fMRI scans, including 505 individuals with ASD and 530 neurotypical control subjects. The data were collected from 17 different imaging sites, each with its own scanning protocol. The dataset has undergone various preprocessing steps, including motion correction, spatial normalization, and noise reduction, to ensure uniform data quality and comparability across different sites. However, different imaging sites had different default fMRI scanners; therefore, Repetition Time (TR), Echo Time (TE), and flip angle degree are varied across sites. The subset of scans with TR of 2000 (ms) from the ABIDE-I dataset has been extracted. Thus, for this study, we have only used data samples collected from 9 out of 17 sites, resulting in 236 ASD samples, 276 typically developing samples. The subjects were then randomly split into training and testing sets. The training and testing sets consisted of 231 control and 235 neurodivergent samples and 35 and 41 samples, respectively. In Figure 2, phenotypic data distributions for the studied data could be found. It could be noted that there are a higher number of male samples than females in both typically developing and neurodivergent subgroups.

### 3.2. Data Preprocessing

**Figure 3:**
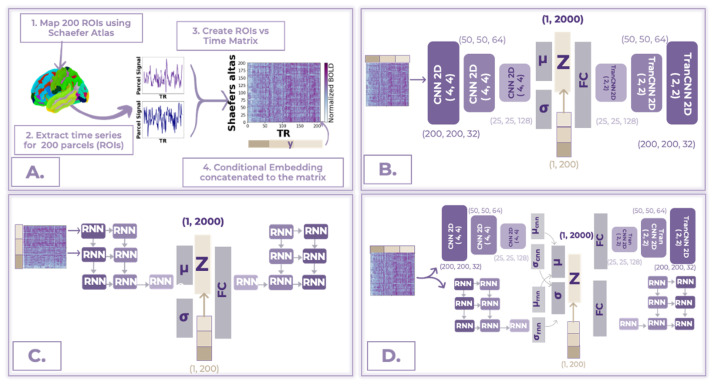
Details on different structures of the model architecture for our FC analysis with fMRI data. (**A**) The overall signal processing framework. (**B**) CNN CVAE. (**C**) RNN CVAE. (**D**) Hybrid CVAE with CNN and RNN in parallel.

Schaefer’s 200-parcel functional deterministic atlas has been used for brain parcellation of the original fMRI scans, which divided the cerebral cortex into 200 distinct, non-overlapping regions based on the derived functional connectivity patterns (Figure 3A) [47]. The resulting 200 parcels are distributed across both hemispheres and cover the entire cortex. Time-series data were extracted from each of the 200 parcels, resulting in a 2D matrix consisting of signals from 200 parcels with 200 time steps (TR=2000 ms). As the length of scans varied across imaging sights, each scan was augmented into multiple samples using a sliding window of 200 time steps with a step size of 10 applied to each voxel per time matrix. The sliding window was then applied to each sample in training and testing subsets, resulting in disjoint 3472 neurotypical and 2973 neurodivergent samples for the training set and 364 and 364 samples for the testing set. The testing and training fMRI splitting, described in Section 3.1, have not been mixed during data augmentation to ensure fairness. Finally, the parcel versus time matrices were normalized to the range of 0 to 1.

### 3.3. Variational Autoencoder (VAE)

The Autoencoder (AE) is a type of neural network architecture commonly employed for capturing low-dimensional representations of fMRI data. AE comprises an encoder and a decoder [48]. The encoder part of the AE transforms the input data into a set of low-dimensional latent variables, and the decoder part subsequently reconstructs those latent variables into the original data space [48]. During training, the encoder and decoder aim to minimize the reconstruction error between the input data and the reconstructed output [48]. A unique subtype of AEs is the Variational Autoencoder (VAE). Similar to the AE, the VAE also consists of an encoder and a decoder, but the encoder maps the input data to a set of latent variables that are assumed to be drawn from a prior distribution. The decoder randomly samples from the latent distribution and learns to map these latent variables back to the original data space to reconstruct the sample. Sampling from a learned latent space and decoding these latent features into the original data space allows for the generation of new data samples.

In our study, the VAE is deployed as a deep generative model using different architectures of the encoder g(x;ϕ) and the decoder f(z;θ). The encoder learns to compress the high-dimensional input (parcels versus time matrix) x into lower-dimensional latent representations z, and ϕ and θ are both hyperparameters of the networks. The VAE aims to learn a model for the true data distribution, denoted by p(z,x). The latent space dimensionality is denoted as *d* (i.e., $z\in R^{d}$). The variational posterior distribution is denoted by q(z,x), which is an approximation of the true posterior. The network is trained using the Evidence Lower Bound (ELBO) loss, consisting of the reconstruction and KL divergence terms. The reconstruction term aims to ensure that the VAE can accurately reconstruct the input data, which is represented as the expected negative log-likelihood logp(x|z), where p(x|z) is modeled by the decoder part of the VAE. The KL divergence term is used to make the variational posterior distribution, q(z|x), as close to the prior distribution, p(z), as possible.

The ELBO loss, denoted as $L_{ELBO}\left(x\right)$, can be written as:

(1) $$\begin{array}{cc} L_{ELBO}\left(x\right) & =E_{q(x,z)}\left[\log\frac{p(z,x)}{q(z|x)}\right]\\ & =E_{q(z,x)}\left[\log p\left(x|z\right)+\log p\left(z\right)-\log q\left(z|x\right)\right]\\ & =E_{q(z,x)}\left[\log p\left(x|z\right)\right]-D_{KL}\left[q\left(z|x\right)|p\left(z\right)\right], \end{array}$$

During training, the encoder network g(x;ϕ) models the variational posterior distribution q(z|x). The encoder outputs the parameters of a Gaussian distribution, $\tilde{\mu }$ and $\log{\tilde{\sigma }}^{2}$, which represent the mean and log-variance of the latent space distribution, respectively. Sampling from q(z|x) allows us to generate new data samples similar to those present in the training data distribution.

### 3.4. Conditional VAE

We propose using a CVAE for a more controlled fMRI sample reconstruction. The CVAE is an extension of the VAE that allows the generation of data samples conditioned on certain attributes or labels [15]. In our CVAE design, both the encoder and decoder receive additional input variables, which is an embedding (denoted as y) containing age, sex (M or F), and subgroup (TD or ASD) labels, with the assumption that all conditions are statistically independent of each other. This can be viewed as concatenating the embedding to the input of the encoder x and the input of the decoder z. The changes made in comparison to the generative process of a VAE can be understood as introducing an identity function with respect to y into the model. In the CVAE, the encoder learns to extract hidden representations of an image x while taking into account conditional variables y (represented by the distribution q(z|x,y)). The decoder then translates this data representation in the form of (z,y) to the input space (i.e., p(x|z,y)).

Specifically, the generative process of the CVAE takes the form

(2) $$\begin{array}{cc} \left({\tilde{\mu }}_{xy},\log{{\tilde{\sigma }}_{xy}}^{2}\right) & =g(z,y;\phi ),\\ q(z|x,y) & =N\left(x;\mu_{xy},\mathrm{diag}\left(\sigma_{xy}^{2}\right)\right) \end{array}$$

And the ELBO loss can then be written as:

(3) $$\begin{array}{cc} L_{ELBO}\left(x|y\right) & =E_{q(z,x,y)}\left[\log\frac{p(z,x|y)}{q(z|x,y))}\right]\\ \; & =E_{q(z,x,y)}\left[\log p\left(x|z,y\right)+\log p\left(z|y\right)-\log q\left(z|x,y\right)\right], \end{array}$$

In the CVAE model, the reconstruction of a sample is dependent on the given set of input conditions. To generate a TD-like output for an atypical sample, the conditional variable must be adjusted to a control condition while retaining the remaining conditions unchanged. Consequently, when calculating the discrepancy between the atypical input and the reconstructed output, the difference is assumed to be solely attributed to the modified conditions. This ensures that the identified divergence depends exclusively on the altered conditional variable.

### 3.5. Experimental Setup

Three commonly used VAE architectures in the fMRI domain were trained to learn a compact representation of the data from neurotypical control fMRI samples. A Convolutional Neural Network (CNN) variational autoencoder, Recurrent Neural Network (RNN) variational autoencoder, and a hybrid of CNN and RNN VAEs in parallel (Figure 3). For all CNN VAEs in this study, the CNN encoder consisted of three convolution layers with 32, 64, and 128 filters, respectively, followed by a fully connected layer. Subsequently, the CNN decoder comprised transposed convolution layers with 128, 64, and 32 filters, followed by a fully connected layer. Batch normalization and the leaky ReLU activation functions were utilized. The RNN encoder contained three unidirectional Long-Short-Term Memory (LSTM) layers followed by a fully connected layer. Decoder, respectively, consisted of a fully connected layer followed by three LSTM layers as well. Finally, the parallel structure model was built as a combination of those CNN and RNN structures in parallel. Latent features are fused using element-by-element multiplication. A more detailed summary of the structures of VAEs can be found in Figure 3. All three VAEs have 2000 latent features extracted by the encoding part (d=2000), and the latent space was modeled using a mixture of Gaussian assumptions. Furthermore, all VAEs were optimized using the Adam algorithm with a learning rate of 0.0001. In the context of the CVAE, all the architectures of the models remain the same; however, the phenotypic data embedding is incorporated by concatenating it with both the input of the encoder and the input of the decoder. The embedding dimensionality is specifically set to 200, allowing for concatenation as another parcel feature to the input matrix, resulting in a total dimensionality of 201×200. Concatenation to the latent vector z resulted in the dimensionality of 2200. It is important to note that for the training of VAEs, only a neurotypical sample has been used; however, due to the conditional embedding, the CVAE allows for training on both neurotypical and neurodivergent samples. All of the experiments that are reported in this paper were performed on the server that contains an NVIDIA RTX 3090 running CUDA version 10.2 and PyTorch 1.13.1+cu117 [49]. We believe that this is the first study in the fMRI domain comparing different encoding and decoding architectures.

### 3.6. VAE Performance Evaluations

Evaluation of VAE performance consisted of analysis of the reconstruction of the neurotypical samples, analysis of latent space features, and analysis of the regeneration abilities of the decoder.

Upon completion of the training, assessment of the VAE and CVAE reconstruction abilities involved three evaluation methods. The cosine similarity score was computed to capture the overall resemblance between the input and the reconstructed output. However, cosine similarity does not explicitly account for positional information. Thus, Pearson’s correlation coefficient (*R*, PCC) was additionally calculated for the validation subsets of the data. Finally, the difference between the input and decoded output was evaluated through L1 (Mean Absolute Error (MAE)). L1 quantified the average absolute difference between the reconstructed BOLD signal intensity and the intensity of the original signal. To compute the L1 error, we leveraged the validation samples of the subgroup present during the training phase. We believe that a combination of these metrics will help us quantify the ability of VAEs and CVAEs to reconstruct samples from lower-dimensional data within the validation dataset.

To assess the encoding abilities of each model, we encode both populations and conduct a comparative analysis of their latent representations. To determine the statistical significance of the differences in the encoding feature, a two-sided *t*-test is employed (p<0.05). The null hypothesis is that the mean of the neurotypical subgroup is equal to the mean of the neurodivergent. It is believed that the optimal encoder architecture will have a pronounced distinction in the latent space, meaning that the encoder learned to extract meaningful features from the input samples. Consequently, our objective is to reject the null hypothesis in favor of the alternative hypothesis, which is that the mean latent representations of the TD and ASD groups are different.

Evaluating the performance of accuracy of synthetic data outputted by VAEs poses a significant challenge, especially when the ground-truth effects are unknown in real data. Therefore, to provide an initial assessment of atypical pattern detection, we calculate L1 of synthetic samples. In the context of VAE experiments, where the model is trained on TD samples only, we formulate a hypothesis that the L1 error would be more pronounced when reconstructing ASD validation samples in comparison to the TD validation samples. For the CVAE experiments, where model architecture accommodates training on both TD and ASD samples, synthetic outputs were generated for the ASD validation dataset with target conditional embedding of TD samples. Consequently, the L1 error is computed between the input ASD samples and the synthetically generated outputs.

### 3.7. Functional Connectivity Analysis

In this study, we conducted FC analysis of the ASD subgroup alongside FC analysis for female and male populations within the ASD group. The FC analysis was performed using trained VAEs and CVAEs in three steps.

**Figure 4:**
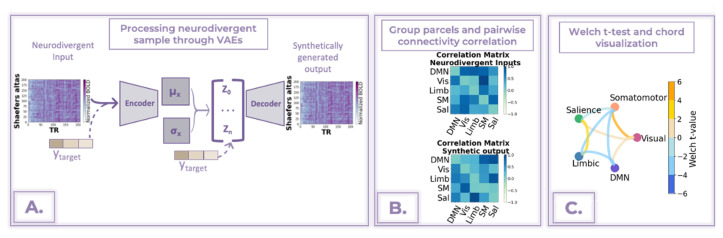
Summary of functional connectivity analysis steps. (**A**) Process neurodivergent samples from the validation subset through VAE or CVAE. Adjust the condition to the target in CVAE. (**B**) Compute pairwise connectivity between networks. (**C**) Perform a two-sided Welch *t*-test and visualize statistically significant results using a chord diagram.

In VAE experiments, we first processed each neurodivergent sample from the validation subset through all three architectures. We hypothesized that since VAEs were trained to reconstruct neurotypical samples only, the output of the neurodivergent sample from the decoding process would resemble the features of the training data (Figure 4A). Next, we grouped the brain parcels into five prominent brain networks—the Default Mode Network (DMN), Limbic, Visual, Somatomotor, and Salience. Due to limitations in Schaefer’s atlas, we could only analyze connectivity within these five networks. We then calculated pairwise connectivity using Pearson correlation coefficients between these networks (Figure 4B). The resulting averaged correlation matrices were then subjected to a two-sided Welch *t*-test to compare interconnectivity within networks between the two subgroups. Statistically significant results (p<0.05) were then visualized using chord diagrams. A negative Welch t-value indicated that the mean of the neurodivergent input was lower than that of the neurotypical-like synthetically generated group, while a positive Welch t-value suggested that the mean of the input group was higher than the generated group. As depicted in Figure 4C, the color of the connecting line between the outer circles of the chord diagram corresponds to the Welch t-value. In this representation, blue shades indicate negative t-values (lower connectivity), while yellow hues correspond to positive t-values (higher connectivity).

For the CVAEs, the training data included both neurodivergent and neurotypical data, which allows for a more targeted generation of the synthetic output. The overall steps for FC with CVAEs were similar to those with VAEs, but the input embedding of the condition was adjusted to the desired output. For instance, if the input sample was a female with ASD, 12 years old, the embedding was adjusted to generate a neurotypical-like female, 12 years old, sample. The remaining FC analysis steps—grouping parcels, calculating pairwise connectivity, conducting two-sided Welch *t*-tests, and visualizing chord diagrams—are the same as with VAEs.

To explore sex-related neurodivergence, we performed separate analyses for female and male samples from the validation dataset. To assess the influence of the conditions on the FC results, we calculate cosine similarity between VAE and CVAE pairwise correlation matrix between networks (Figure 4B). We believe that the cosine similarity score should be higher for CVAE than VAE, indicating reduced sex-related bias.

## 4. Results

### 4.1. VAE Performance Evaluations

**Figure 5:**
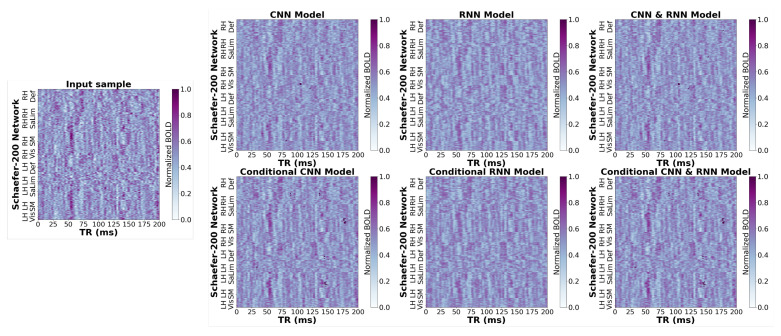
Sample reconstruction of parcels vs. time matrix for a neurotypical control sample from validation subset. LH: Left Hemisphere, RH: Right Hemisphere, Vis: Visual, SM: Somatomotor, Lim: Limbic, Sal: Salience, Def: Default.

**Figure 6:**
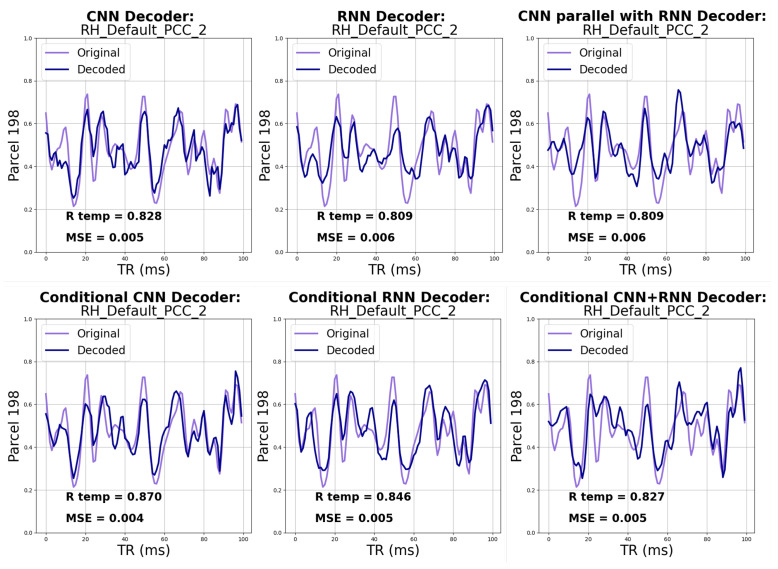
Sample reconstruction of one parcel for the neurotypical control sample from validation subset. PCC and MSE are also stated for the displayed parcel reconstruction.

As detailed in Section 3.6, we begin by evaluating the reconstruction performance of all VAEs and CVAEs. Upon visual inspection of Figure 5, we observe that all models have adeptly learned to reconstruct the data from the low-dimensional representation. In Figure 6, one can observe the decoded signal from one parcel of the validation sample, and the decoded signal closely follows the input signal, demonstrating a high level of reconstruction. Additional quantitative results are summarized in Table 1 and Table 2. It i worth highlighting that integrating conditional variables into the models has increased the accuracy in reconstructing latent features, as indicated by both the cosine similarity and PCC metrics. The CNN architecture has outperformed other architectures in terms of reconstruction across both the VAE and CVAE experiments as evidenced by the highest PCC scores in Table 1 and Table 2. Moreover, the increase in PCC by adding conditional embedding to the CNN model (9.37%) is higher compared to the increases in other models (4.54% in RNN and 3.18% in CNN+RNN).

**Figure 7:**
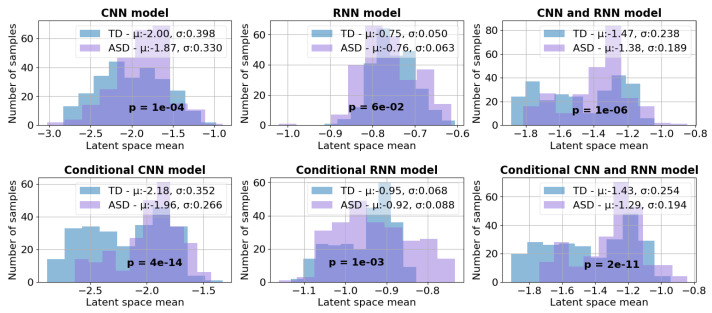
Summary of mean distribution of the latent space for validation subsets for each model. T-test significance is also reported on each of the subplots.

To evaluate the encoding capabilities of each model, a comprehensive analysis was conducted on both neurotypical and neurodivergent samples from the validation dataset. Figure 7 depicts the resulting means of latent distribution. Notably, among the VAE models, the CNN architecture and the hybrid CNN with RNN models exhibit statistically significant differences in their latent features between affected and unaffected samples. Therefore, the models have successfully learned to extract meaningful features from the input data. As anticipated, adding conditional embedding to the models resulted in a higher degree of separation within the latent space than unconditional models. All the CVAE models display statistically significant differences in latent space between the two subgroups.

To further assess the performance of VAEs, we conducted a preliminary evaluation of atypical pattern detection by calculating the reconstruction error on both neurotypical and neurodivergent samples from our validation datasets, summarized in Table 1. The reconstruction L1 error for the ASD validation set is higher than that of the TD set. This difference implies that VAEs can reconstruct ASD samples in a manner that makes them resemble TD samples. For the CVAEs, we conducted a similar analysis. Given that the CVAE was trained on both ASD and TD samples, our approach involved computing the reconstruction L1 error for the ASD samples first. Subsequently, we compared this with the synthetically generated outputs, employing a target conditional embedding based on a TD sample. The results, presented in Table 2, show that the construction error for the synthetic samples exceeds that of the reconstructed ASD samples. This disparity serves as an indication that the conditioning mechanism is effective in detecting certain divergences within the data.

### 4.2. Functional Connectivity Analysis

**Figure 8:**
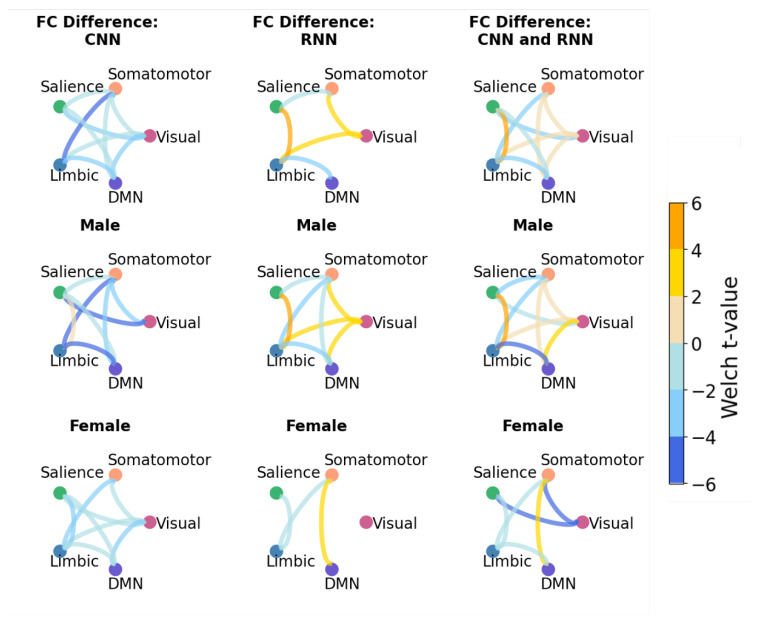
Statistically significant results of FC analysis presented as the chord diagram from VAE experiments (two-sided Welch’s, p<0.05). The bluish color of the lines indicates lower connectivity, while yellowish colors represent higher connectivity of ASD samples compared to neurotypical-like synthetic samples. The top row displays combined results for both female and male populations, the middle row focuses on the male population only, and the bottom row pertains to female samples.

**Figure 9:**
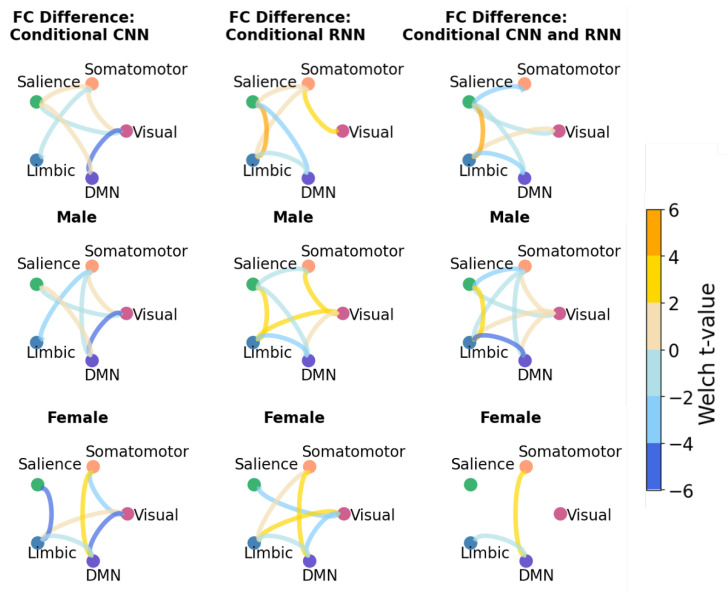
Statistically significant results of FC analysis presented as the chord diagram from CVAE experiments (two-sided Welch’s, p<0.05). The bluish color of the lines indicates lower connectivity, while yellowish colors represent higher connectivity of ASD samples compared to neurotypical-like synthetic samples. The top row displays combined results for both female and male populations, the middle row focuses on the male population only, and the bottom row pertains to female samples.

Figure 8 and Figure 9 present the results of the FC analysis, following the steps outlined in Section 3.7. In Figure 8 and Figure 9, the top row is the connectivity trends for both female and male samples of the testing data, the middle row is the trends for the male population, and the bottom is female. We first highlight results shared consistently across all rows, which indicates the trends that are unaffected by the sex imbalance within the dataset. Subsequently, we summarize the identified patterns in functional connectivity that were affected by this sex bias.

In the VAE experiments (Figure 8, top row), a consistent trend of under-connectivity between the Limbic and DMN networks emerges across all models for the combined male and female populations. This pattern remains evident in both the female and male subpopulations, except for the RNN female results (Figure 8, bottom row). Similarly, CNN and hybrid models identified under-connectivity between the salience and visual networks, which has remained similarly apparent in both male and female populations. Finally, the trend that is found to be common across males subpopulation and females subpopulation (middle and bottom rows) is the under-connectivity between limbic and somatomotor networks.

One notable consequence of the dataset’s bias is exemplified by the consistent trend of over-connectivity between the salience and limbic networks in the male population, which is reversed in females for all of the models (Figure 8, middle and bottom rows). Thus, connectivity between the salience and limbic networks for the combined populations (top row) reveals contrasting outcomes. Furthermore, a noteworthy difference between males and females lies in the connectivity between the somatomotor and DMN networks. In females, the connectivity between the somatomotor and DMN networks exhibits a notably stronger presence in comparison to males. This can be concluded by higher Welch t-values observed within the female population for the connections between the somatomotor and DMN networks.

In the CVAE experiments, some trends are similar to those identified with VAE models. For example, in Figure 9, a trend of under-connectivity between limbic and DMN is apparent for both the male and female populations, with the exception of the CNN model. In RNN-based and hybrid models, the trend of under-connectivity between limbic and DMN in males and females remains true for CVAE experiments. Remarkably, the trend of increased connectivity between the Somatomotor and DMN networks in females, as opposed to males, persists in CVAE experiments as well.

Interpreting the chord plots and discerning the extent to which the CVAE mitigated sex-related influences presents a challenge. As outlined in Section 3.4, the identified neurodivergence in the CVAE is expected to have a lower correlation with sex labels compared to the VAE. To measure this, we quantitatively assess the similarity between the pairwise correlations underpinning these chord plots (Table 3). This similarity score revealed that all the conditional models have a higher overlap between male and female neurodivergence compared to the unconditional models. Conditional hybrid model had the highest values for the similarity between female and male pairwise correlation matrices, suggesting the most unbiased FC neurodivergence patterns in relation to sex. However, it is important to note that the CNN model compared to the rest of the models had the highest increase in similarity by adding conditional embedding, which is indicative of the fact that CNN layers are particularly sensitive to the inclusion of conditional embedding.

## 5. Discussion

In this study, we investigated the application of generative models to FC analysis in the context of ASD with fMRI data. Our exploration began with a comprehensive assessment of the reconstructive abilities of various VAE architectures, using neurotypical samples as the input data.

We believe that the CNN-based VAE and CVAE are more effective in reconstruction and conditional generation of synthetic data. This conclusion has been derived from the combination of the metrics and results provided in Section 4. First and foremost, cosine similarity and PCC measures for CNN VAE and CNN CVAE reconstruction are higher compared to the other models. Even though introducing phenotype data has improved both reconstruction in higher dimensional space and discrimination in lower-dimensional space for all of the models, it i worth highlighting that the CNN model has demonstrated superior reconstruction performance with conditioning, as evidenced by the highest observed increase in PCC by comparing Table 1 and Table 2. Secondly, in Table 3, the CNN model stands out with the highest increase in similarity between female and male connectivity, almost doubling the improvement seen in other models. These findings collectively indicate that the CNN model exhibits heightened sensitivity to conditioning mechanisms in comparison to other models. One plausible explanation for this could lie in the way the condition is introduced to the model. In all models, conditional embedding is concatenated as another feature, resulting in the absence of explicit temporal ordering within conditional embeddings. Consequently, CNN demonstrated superior performance in handling this conditioning mechanism when contrasted with the RNN model. We interpret this as it is more effective for the VAE to model spatial patterns rather than temporal ones. We believe that unconditional CNN in parallel with RNN is better for classification applications.As shown in Figure 7, the degree of separation for the unconditional hybrid model is higher than the other models. Secondly, the addition of conditional information to the hybrid model resulted in the most unbiased results in relation to sex labels results, as indicated by the highest values in Table 3. Nevertheless, the hybrid model has reduced sex-related bias the most, the results were accompanied by poorer reconstruction performance compared to the CNN-based model.

To provide initial validation for the decoder architectures, we calculated the Mean Absolute Error for the reconstruction of the subgroup that was present during the training and the new sample subgroup. VAEs had higher reconstruction errors for ASD samples compared to TD samples, indicating their ability to model ASD samples resembling TD ones. For CVAEs, which were trained on both ASD and TD samples, we computed reconstruction loss for ASD samples. Comparing this loss of synthetically generated outputs using a TD-based target conditional embedding, we found higher reconstruction errors for synthetic samples. This finding also suggests the conditioning mechanism effectively detects neurodivergence and can make the generation process more targeted. Moreover, in comparison to the work of Kim et al. [22], our VAE models demonstrate better performance in data reconstruction, showcasing a lower range of reconstruction errors ranging from 0.06 to 0.07, while Kim et al. reported a range of 1.2 to 1.7.

Next, we proceeded further to FC analysis with trained VAEs and CVAEs. We consistently identified under-connectivity between the limbic and DMN networks across most VAE experiments, which is consistent with previous findings in the literature [38]. The trend of higher connectivity between salience and limbic networks in the male population compared to female has been identified by all VAEs and CVAEs. In the study by Green et al. [42], where the studied group consisted predominantly of the male population, they concluded that male individuals tend to exhibit over-connectivity between salience and limbic networks. However, we extend these findings and show this trend does not hold true in the female population.

One of the findings in the previous literature is that males tend to have decreased under-connectivity with the DMN network compared to females [44]. Based on our analysis, both the VAE and CVAE revealed this pattern as well, specifically between DMN and somatomotor networks. Due to the limitations of Schaefer’s atlas, we have focused on exploring network connectivity. However, in future works, we aim to expand the investigation to other atlas configurations and within network analysis. All of the above are consistent with the connectivity patterns that have been reported previously in other literature, summarized in Section 2.4.

It was hypothesized that adjusting conditional embedding would reduce sex-related bias in the models and potentially result in sex-independent FC. By evaluating the pairwise connectivity matrix overlap between female and male subgroups, it is concluded that patterns discerned through CVAE have reduced correlation with sex labels. We believe the remaining differences shown in the chord plot between male and females in CVAE experiments are primarily due to the age difference and diverse nature of the disorder.

In recent years, many studies have explored the capabilities of generative models (including VAEs, GANs, and Diffusion flow models) in the medical domain. However, many models are found to struggle with at least one of the following: high-quality outputs, mode coverage, sample diversity, and computational costs [50]. VAEs are probabilistic models, which makes them well-suited for modeling and generating complex distributions. As shown in this paper, VAEs can learn the underlying probability distribution of the input data, allowing for probabilistic sampling and interpolation. However, as stated in previous works, VAEs tend to suffer from comparatively low quality in generation compared to GANs or Diffusion flow models [50]. Therefore, our future work will also investigate different generative frameworks to improve the quality of generated samples and develop methods for assessing them.

## 6. Conclusions

This paper presents a novel approach to FC analysis of fMRI data using a generative model such as the VAE. We also attempted to study if introducing additional phenotype data to the model would reduce bias and increase the generalizability of the FC analysis. Our main finding includes that the CNN-based model has been shown to be the most effective architecture for the FC analysis, as it showed superior performance in reconstruction with and without conditional information. We show that introducing phenotypic data to the model generally improves reconstruction performance and reduces bias in FC analysis. Conditioning of the CNN model has shown to have the most effect on the results; however, the CNN model parallel with RNN has shown to be the least biased with respect to sex labels.

## Figures and Tables

**Table 1:** Summary of reconstruction performance of VAE experiments: cosine similarity scores and PCC for the neurotypical samples in the validation dataset. The average L1 reconstruction error for both neurotypical and neurodivergent samples within the validation dataset is presented.

Model	Cosine Similarity	PCC	L1 TD	L1 ASD
CNN	0.9930	0.6551	0.0693	0.0781
RNN	0.9817	0.6105	0.0728	0.0819
CNN and RNN	0.9820	0.6356	0.0717	0.0803

**Table 2:** Summary of reconstruction performance of CVAE experiments: cosine similarity scores and PCC for the neurotypical samples in the validation dataset. Additionally, the average L1 reconstruction error for validation neurodivergent samples and synthetically generated neurotypical-like samples.

Model	Cosine Similarity	PCC	L1 ASD	L1 TD${}_{\mathrm{synthetic}}$
Conditional CNN	0.9961	0.7165	0.0643	0.0733
Conditional RNN	0.9818	0.6382	0.0681	0.077
Conditional CNN and RNN	0.9825	0.6558	0.0687	0.0778

**Table 3:** Similarity between male and female FC pairwise correlations for VAE and CVAE experiments.

Model Architecture	Unconditional FC Similarity	Conditional FC Similarity
CNN	0.35	0.70
RNN	0.66	0.80
CNN parallel with RNN	0.78	0.85

## Data Availability

This study used publicly available dataset that can be found at https://fcon_1000.projects.nitrc.org/indi/abide/ accessed on 18 August 2023.

## Abbreviations

The following abbreviations are used in this manuscript:

ASD	Autism Spectrum Disorder
TD	Typically Developing
FC	Functional Connectivity
fMRI	functional Magnetic Resonance Imaging
BOLD	Blood-Oxygen-Level-Dependent
CNN	Convolutional Neural Network
RNN	Recurrent Neural Network
VAE	Variational Autoencoder
CVAE	Conditional Variational Autoencoder
DMN	Default Mode Network
PCC	Pearson’s Correlation Coefficient
